# Automated measurement of malaria parasitaemia among asymptomatic blood donors in Malawi using the Sysmex XN-31 analyser: could such data be used to complement national malaria surveillance in real time?

**DOI:** 10.1186/s12936-022-04314-3

**Published:** 2022-10-25

**Authors:** Michael Kayange, Bridon M’baya, Talent Hwandih, Jarob Saker, Thérèsa L. Coetzer, Marion Münster

**Affiliations:** 1 National Malaria Control Programme, Ministry of Health, Lilongwe, Malawi; 2 Malawi Blood Transfusion Service, Blantyre, Malawi; 3 Sysmex Europe SE, Bornbarch 1, 22848 Norderstedt, Germany; 4 Faculty of Health Sciences, Wits Research Institute for Malaria, University of the Witwatersrand, Johannesburg, South Africa

**Keywords:** Malaria surveillance, Asymptomatic blood donors, Malawi, XN-31, Automated malaria detection, Complementary data

## Abstract

**Background:**

The recent worldwide increase in malaria cases highlights the need for renewed efforts to eliminate malaria. The World Health Organization advocates that malaria surveillance becomes a core intervention. Current methods to estimate the malaria burden rely on clinical malaria case reports and surveys of asymptomatic parasite infection mainly from children  < 5 years. In this study the hypothesis was that screening blood donors for malaria parasites would provide real-time information on the asymptomatic reservoir of parasites in the adult population and mirror other surveillance data.

**Methods:**

This study was conducted in Malawi, a high malaria burden country, at the Malawi Blood Transfusion Service, which collects blood units at donation sites countrywide. A secondary analysis was conducted on data obtained from a prior Sysmex XN-31 analyser malaria diagnostic evaluation study utilizing residual donor blood samples. XN-31 malaria results, donor age, sex, geographical location, and collection date, were analysed using standard statistical methods.

**Results:**

The malaria parasite prevalence in blood donors was 11.6% (614/5281 samples) increasing seasonally from December (8.6%) to April (18.3%). The median age was 21 years and 45.9% of donors were from urban areas, which showed a lower prevalence compared to non-urban regions. The Central administrative region had the highest and the Northern region the lowest malaria parasite prevalence. The donors were predominantly male (80.2%), 13.1% of whom had malaria parasites, which was significantly higher (p < 0.0001) than for female donors (7.4%). Multivariable logistic regression analysis showed that age, location, and collection month were significant predictors of malaria positivity in males, whereas in females only location was significant. There was no gender difference in parasite density nor gametocyte carriage.

**Conclusions:**

This study demonstrates the powerful utility of screening blood donors for malaria parasites using the XN-31, which not only improves the safety of blood transfusion, but provides valuable complementary surveillance data for malaria control, especially targeting males, who are generally excluded from periodic household surveys. Blood donations are sourced countrywide, year-round, and thus provide dynamic, real-time information on the malaria burden. Furthermore, the XN-31 identifies the asymptomatic human reservoir of infectious gametocytes, which must be targeted to eliminate malaria.

## Background

Malaria, despite being preventable and treatable, with an estimated 241 million cases in 2020 in 85 malaria-endemic countries, continues to be a major contributor to the global health burden [1]. Notwithstanding the great progress that has been made over the past two decades, a concerted global, yet focused, effort is required if the vision of the World Health Organization (WHO) and global malaria community of a malaria-free world is ever to be attainable [2]. To this end, the WHO Global Technical Strategy (GTS) for malaria has outlined 3 pillars to guide global efforts towards elimination. These are (1) ensure universal access to malaria prevention, diagnosis and treatment; (2) accelerate efforts towards elimination and attainment of malaria-free status and (3) transform malaria surveillance into a core intervention. Despite the current upswing in malaria cases from 224 million in the 2015 GTS baseline, the GTS goals and targets remain unchanged in the 2021 update of this document [3]. As the COVID-19 pandemic has contributed significantly to this reversal of progress, the need for increased resilience in health care systems to withstand such unforeseen disruptions has been added as a guiding principle in support of the ultimate goal of malaria elimination [3].

Key elements of an effective malaria surveillance strategy are to systematically and continuously collect high quality data on the burden of malaria and to integrate these findings into national health information systems [3]. These data are used to assess the effects of intervention measures, analyse trends, track changes in malaria prevalence and enable efficient planning of resource allocation for malaria control programmes. The surveillance method should ideally target groups who are easily accessible and representative of the population at risk of malaria, and also cover all geographical areas within an endemic country to capture the heterogeneity of malaria prevalence. In addition to active case surveillance, the reservoir of asymptomatic malaria carriers should be identified, including individuals harbouring gametocytes, since these are transmitted to the mosquito vector and enable the parasite to continue its lifecycle. A surveillance programme must be sustainable and in malaria-endemic, resource-poor countries, it requires cost-effective measures and simple logistics to ensure optimal use of human resources and to remain within budget constraints.

Whilst malaria is fundamentally viewed as a vector borne parasitic disease, transmitted by the bite of infected female *Anopheles* mosquitoes, it is also readily transmissible via transfusion of infected blood [4–7]. The actual risk of clinical malaria in recipients of malaria-infected blood transfusions in malaria-endemic countries is however largely unknown [8], notably as distinction between transfusion and environmentally acquired malaria parasites, an ever-present risk, requires parasite genotyping which is not routinely available. However, as the global malaria burden declines, and elimination continues to be embraced as a global goal, malaria transmitted via transfusions cannot be ignored. Achieving malaria elimination requires targeting the human reservoir of infection, including those with asymptomatic infection, such as healthy blood donors. Although the risk of transfusion transmitted malaria (TTM) cannot be entirely eliminated in higher prevalence areas, even with the most sensitive screening tests available, screening blood products for malaria could at least reduce this risk at community level. This is particularly important as the largest consumers of blood products in malaria-endemic countries in sub-Saharan Africa, are pregnant women and young children, who are also the most vulnerable to poor clinical outcomes, should TTM occur. Whilst the reference to malaria prevention in the first GTS pillar highlights vector control and chemoprevention, reducing the occurrence of TTM also could and should be considered as a key pillar of prevention, notwithstanding the complexity of balancing blood safety with adequacy of supply in malaria-endemic countries.

The Sysmex XN-31 analyser (XN-31), a new higher diagnostic sensitivity technology for automated malaria detection, was recently evaluated at the Malawi Blood Transfusion Service (MBTS) with the specific objectives to determine the sensitivity and specificity of XN-31 for malaria screening and to estimate true malaria parasite prevalence in the MBTS donor pool. The study confirmed that the XN-31 had excellent sensitivity and specificity, revealing an overall malaria parasite prevalence of 11.6% amongst blood donors, in contrast to the 6.5% detection rate for thick smear microscopy, the method in routine use for malaria screening at the time of the study [9]. Malawi is a high malaria burden country, with perennial transmission and the entire population at risk of infection, almost exclusively due to *Plasmodium falciparum* [1] although mixed infections and non-falciparum mono-infections do occur [10]. However, significant variability in malaria positivity rates was observed based on geographical location of blood collection sites. This observation led to the hypothesis that implementing XN-31 as a malaria screening tool within blood banks, in addition to reducing TTM risk, could be a complementary source of surveillance data, generated in real-time, to support national malaria control programmes. Such data would serve to continuously track trends in the size and location of the reservoir of asymptomatic parasitaemia within the community in real-time, as household surveys by virtue of their logistic complexity, are episodic in nature.

The objective of this secondary data analysis was to provide a detailed analysis of the donor demographics and assess whether the observed patterns and trends mirror those obtained from standard malaria surveillance activities, such as household malaria indicator surveys (MIS). The ultimate aim was to show that donor malaria screening could be used as a source of real-time surveillance data, in line with the third GTS pillar, namely, to transform malaria surveillance into a core intervention. Understanding the nature of the reservoir of malaria parasites in asymptomatic individuals is a key component along the path towards malaria elimination.

## Methods

### Study setting

Malawi is a high malaria burden country with perennial transmission and the entire population at risk of infection. This study was conducted at MBTS, a centralized service which collects blood units at numerous donation sites countrywide, covering 27 of the 28 administrative districts (excluding Likoma island). There are 4 static (permanent) donation sites in Blantyre, Balaka, Lilongwe and Mzuzu which are in the Southern, South-Eastern, Central and Northern regions of Malawi respectively and mobile units in all 27 districts. Blood samples were transported to one of these 4 permanent sites and then to the central laboratory in Blantyre for centralized blood-borne pathogen screening and blood typing.

### MBTS blood donor recruitment criteria

All blood donations collected by MBTS are from voluntary unpaid donors. Pre-donation screening consists of two stages: (1) educating blood donors on the eligibility criteria for blood donation aimed at encouraging self-deferral among individuals willing to donate blood and (2) administering a blood donation form which contains the national blood donation acceptability criteria. This helps screen out those who are not eligible to donate blood. The exclusion criteria for blood donation are extensive and include the following: age below 16 or above 65 years; body weight below 42 kg; a feeling of unwellness; engagement in behaviour deemed to increase risk for transfusion transmissible infections (such as tattooing and multiple sexual partners); past medical history suggestive of HIV, hepatitis or syphilis; past or present history of renal, cardiovascular, central nervous system and metabolic disorders such as insulin-dependent diabetes; and haemoglobin below 12.5 g/dl. Only individuals who satisfy the national blood donation acceptability criteria are accepted as blood donors.

Most blood collection (~ 65%) takes place at mobile collection sites. MBTS staff visit donation sites 1–2 weeks prior to the scheduled donation day to sensitize potential donors with regards to the donation process and eligibility criteria. MBTS also provides an incentive to encourage repeat donation through their milestone rewards programme, which entails standardized gifts, comprised of MBTS branded items, such as caps, T shirts and certificates, depending on number of donations made, from the third donation onwards.

### Study aim and design

This study is a secondary data analysis of a prior study [9] that was undertaken to establish the usefulness of XN-31 for malaria screening in a malaria-endemic setting and to establish the true prevalence of malaria parasitaemia amongst blood donors in Malawi. The primary study was a prospective observational study that used residual venous blood samples from donors at MBTS donation sites countrywide for routine blood-borne pathogen screening. Blood tubes were transported to the central laboratory in Blantyre for blood typing and malaria thick smear microscopy. Residual consecutive blood donor samples received on low workload days, were used for XN-31 malaria testing. Demographic data captured included donor age, sex, collection date, collection site and collection centre. The aim of this secondary data analysis was to undertake an in-depth analysis of the original study data in terms of blood donor demographics with particular emphasis on age, sex, geographical location (administrative district based on donation site), malaria status and gametocyte carriage to identify prevalence patterns and trends.

### Sysmex XN-31 analyser automated malaria screening

The measurement principle of XN-31, an analyser essentially identical to its research-use-only predecessor the XN-30, has been detailed elsewhere [11]. Briefly, an absolute and percentage MI-RBC (malaria infected red blood cell) count is generated, including all asexual parasite life stages as well as gametocytes. Samples were measured in the LM (low malaria mode) which has an analytical limit of quantification of 20 parasites/µL (p/µL). XN-31 can detect all *Plasmodium* species causing human malaria. The analyser also provides a concurrent complete blood count measurement for each sample analysed.

### Analysis of XN-31 malaria prevalence rates based on district and weather zones

The XN-31 malaria results were segregated based on the administrative district location of the individual blood collection sites, weather zones as defined by the Department of Climate Change and Meteorological Services, Ministry of Forestry and Natural Resources, Malawi [12] as well as month of collection. Blood donations collected at Blantyre and Lilongwe city collection sites were classified as urban, and the remainder as non-urban.

### Analysis of biological and environmental determinants of malaria prevalence in asymptomatic blood donors

Donor age, sex and geographic location were analysed to ascertain the primary determinants of malaria prevalence.

### Statistical analysis

Data analysis was done using MedCalc^®^ Statistical Software version 19.8 (MedCalc Software Ltd, Ostend, Belgium). A descriptive analysis was done to summarize donor demographic data such as sex and age. Continuous data were expressed as medians and interquartile ranges (IQR) while categorical variables were summarized as percentages. Prevalence was estimated by dividing the number of malaria positive samples by the total sample size. An acceptable minimum sample size for prevalence estimation was set at 30 [13]. Malaria prevalence estimates were calculated and mapped to their respective administrative and weather zones. Bivariate statistics were run to determine significant differences between two independent groups. Mann–Whitney test was used for continuous variables whereas Chi-squared test was used for comparison of two independent proportions. Additionally, a multivariable logistic regression was done to investigate potential determinants of malaria prevalence. Results of the logistic regression model were expressed as odds ratios (OR) with 95% confidence intervals (CI) for each independent variable. For all statistical tests, p values below 0.05 (two-tailed) were considered statistically significant.

## Results

### Malaria screening results

The details of comparative microscopy and XN-31 malaria screening results are described in detail elsewhere [9]. In brief, malaria parasites were observed in 6.5% (341/5281) of samples screened by routine microscopy whereas XN-31 judged 11.6% (614/5281) as malaria positive (MI-RBC present), with a median of 164 p/µL (IQR: 63–448) ranging from 20 to 13514 p/µL.

### Donor demographics

#### Sex

The donor group was comprised of 80.2% (4236/5281) male, 17.4% (918/5281) female and 2.4% (127/5281) for whom this information was not captured.

#### Age

The median age of blood donors was 21 years (IQR 19–28), with 95% of donors between 16 and 47 years, and the oldest 64 years. Female donors were significantly younger (p < 0.001, Mann–Whitney test) with median age of 19 years (IQR 18–25) compared with males (median 21 years, IQR 19–28). There were also differences in age distribution based on collection centre. Donors, whose samples were sent to the Lilongwe blood collection centre were the oldest (median 23 years, IQR 19–31) while donors from Mzuzu were the youngest (median 19 years, IQR 18–22). The median age of donors from Balaka and Blantyre was 20 years (IQR 19–25) and 21 years (IQR 19–27), respectively. Donors from exclusively urban areas (n = 2103, 40%) with median age of 25 years (IQR 20–32) were significantly older (p < 0.001, Mann–Whitney test) compared with 20 years (IQR 18–23) for donors from predominantly non-urban collection sites (n = 3159, 60%).

### Location

#### Analysis of XN-31 malaria prevalence rates based on district, administrative region, and weather zones

**Fig. 1:**
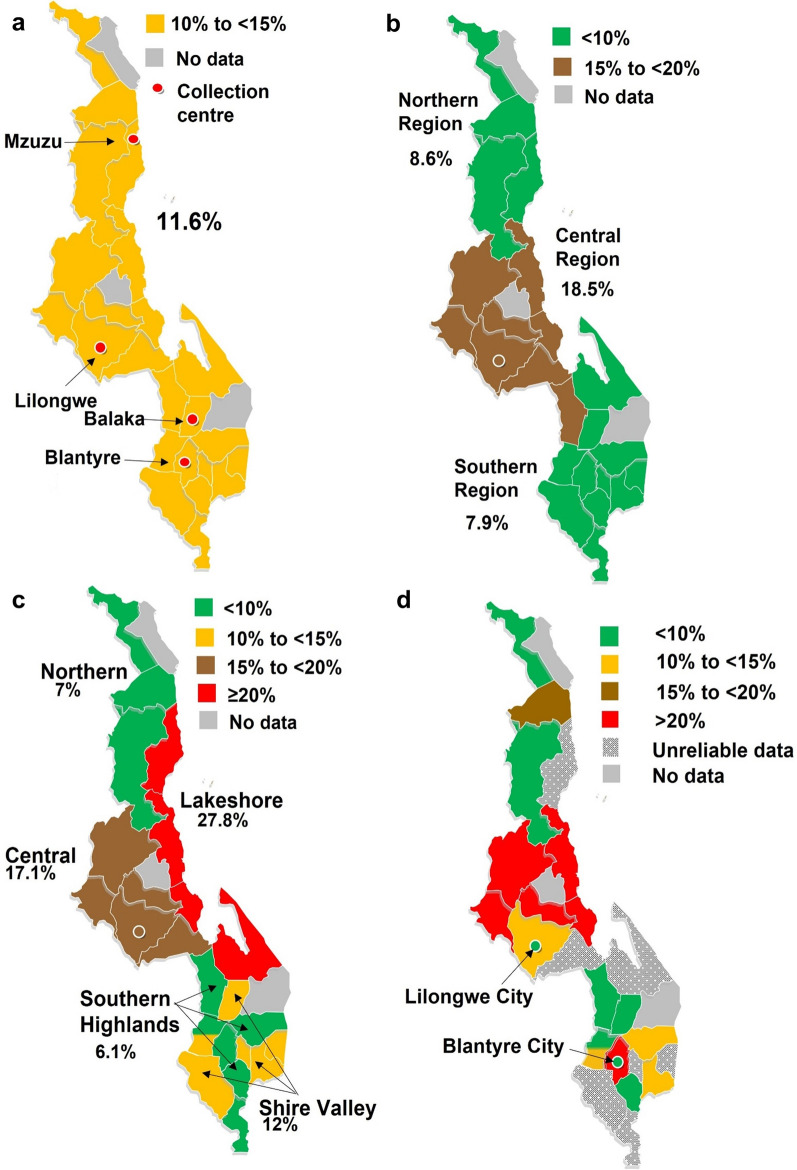
Donor malaria prevalence based on administrative region, weather zone, and administrative district. **a** Overall donor malaria prevalence (11.6%.) The geographic location of the MBTS collection centres Mzuzu, Lilongwe, Balaka and Blantyre are shown, **b** Donor malaria prevalence by administrative region, **c** Donor malaria prevalence by weather zone (weather zones straddle district boundaries, thus this map reflects an approximation only as each district had to be assigned to a single weather zone), **d** Donor malaria prevalence by administrative district. The Lilongwe and Blantyre district prevalence figures reflect the malaria prevalence in non-urban donors with the donors located in Lilongwe city and Blantyre city shown separately. Data from sites with  < 30 samples were deemed unreliable

Blood samples included in this study were from blood donations collected in 24 of the 28 Malawian administrative districts, which are grouped into the Northern, Central and Southern administrative regions. The weather zones are comprised of the northern areas, central areas, lakeshore areas, Southern Highlands, and the Shire Valley. The 5281 samples included in the study comprised ~ 25% of the total donations collected during the study period, with 45.9% coming from urban donors. Although urban donors seem “oversampled”, considering that the Malawi population is 16% urban and 84% non-urban [14], no weighting could be applied in this analysis as the original study was not designed with the intention of using the data for malaria surveillance. Accordingly, comments regarding malaria prevalence have been restricted to the blood donor group. The overall malaria parasite prevalence in urban donors included in this study was 4.1% (115/2338) compared with 18.1% (499/2753) in non-urban donors. The donor malaria prevalence at weather zone, administrative region and district level is shown in Fig. 1 and Table 1, which should be viewed together. Prevalence rates were only calculated for districts with  ≥ 30 samples. Districts were also grouped into arbitrarily chosen prevalence zones based on the district-level donor malaria prevalence as follows: low  < 10%; low-medium  ≥ 10% < 15%; medium–high  ≥ 15% < 20%; high  ≥ 20%. The median MI-RBC count was different (p = 0.0483) in low (0.122 p/µL) and high (0.170 p/µL) prevalence areas. The proportion of infected donors with low level parasitaemia (< 100 p/µL) was higher in regions with low endemicity (43%, 64/147) compared to high prevalence areas (34%, 131/383), but this difference did not reach statistical significance (p = 0.0542).

**Table 1:** Donor malaria prevalence and contribution to donor pool at district level

Weather zone	Administrative district (region)^#^	Donor parasitaemia prevalence	Proportion of donor pool*
Northern	Rumphi (N)	17.0% (15/88)	1.7%
	Mzimba (N)	4.5% (14/311)	6.1%
	Chitipa (N)	3.3% (1/30)	0.6%
	Karonga (N)	No data	0%
	ALL	7.0% (30/429)	8.4%
Central	Dowa (C)	--- (6/14)	0.3%
	Kasungu (C)	32.2% (29/90)	1.8%
	Lilongwe (urban) (C)	7.4% (63/857)	16.8%
	Lilongwe (non-urban) (C)	25.9% (178/686)	13.5%
	Mchinji (C)	28.1% (16/57)	1.1%
	Ntchisi (C)	No data	0%
	ALL	17.1% (292/1704)	33.5%
Lakeshore	Likoma* (N)	No data	0%
	Mangochi (S)	20.0% (42/210)	4.1%
	Nkhotakota (C)	36.6% (30/82)	1.6%
	Nkhata Bay (N)	--- (9/25)	0.5%
	Salima (C)	38.0% (27/71)	1.4%
	ALL	27.8% (108/388)	7.6%
Shire valley	Balaka (S)	5.7% (2/35)	0.7%
	Chikwawa (S)	--- (3/14)	0.3%
	Chiradzulu (S)	--- (0/8)	0.2%
	Mulanje (S)	13.7% (7/51)	1.0%
	Mwanza (S)	11.8% (40/338)	6.6%
	Phalombe (S)	--- (2/4)	0.1%
	ALL	12.0% (54/450)	8.9%
Southern highlands	Blantyre (urban) (S)	3.5% (44/1246)	24.5%
	Blantyre (non-urban) (S)	33.0% (32/96)	1.9%
	Dedza (C)	--- (9/23)	0.5%
	Machinga (S)	--- (0/1)	0.02%
	Neno (S)	7.5% (16/212)	4.2%
	Nsanje (S)	--- (0/2)	0.04%
	Ntcheu (C)	5.7% (5/87)	1.7%
	Thyolo (S)	0.9% (2/234)	4.6%
	Zomba (S)	10.0% (22/219)	4.3%
	ALL	6.1% (130/2120)	41.6%

^*^“Proportion of donor pool” refers to the number of blood donor study samples collected in a particular district, expressed as a percentage of the total number of donor samples included in the study

^#^The contribution to the donor pool by administrative region is as follows: Northern (N) 9%, Central (C) 39%, Southern (S) 52%

^*^Due to inaccessibility, Likoma Island is not an MBTS blood collection site

---Sample size too small (< 30) for reliable prevalence rate calculation

### Donor malaria prevalence trends by month

**Fig. 2:**
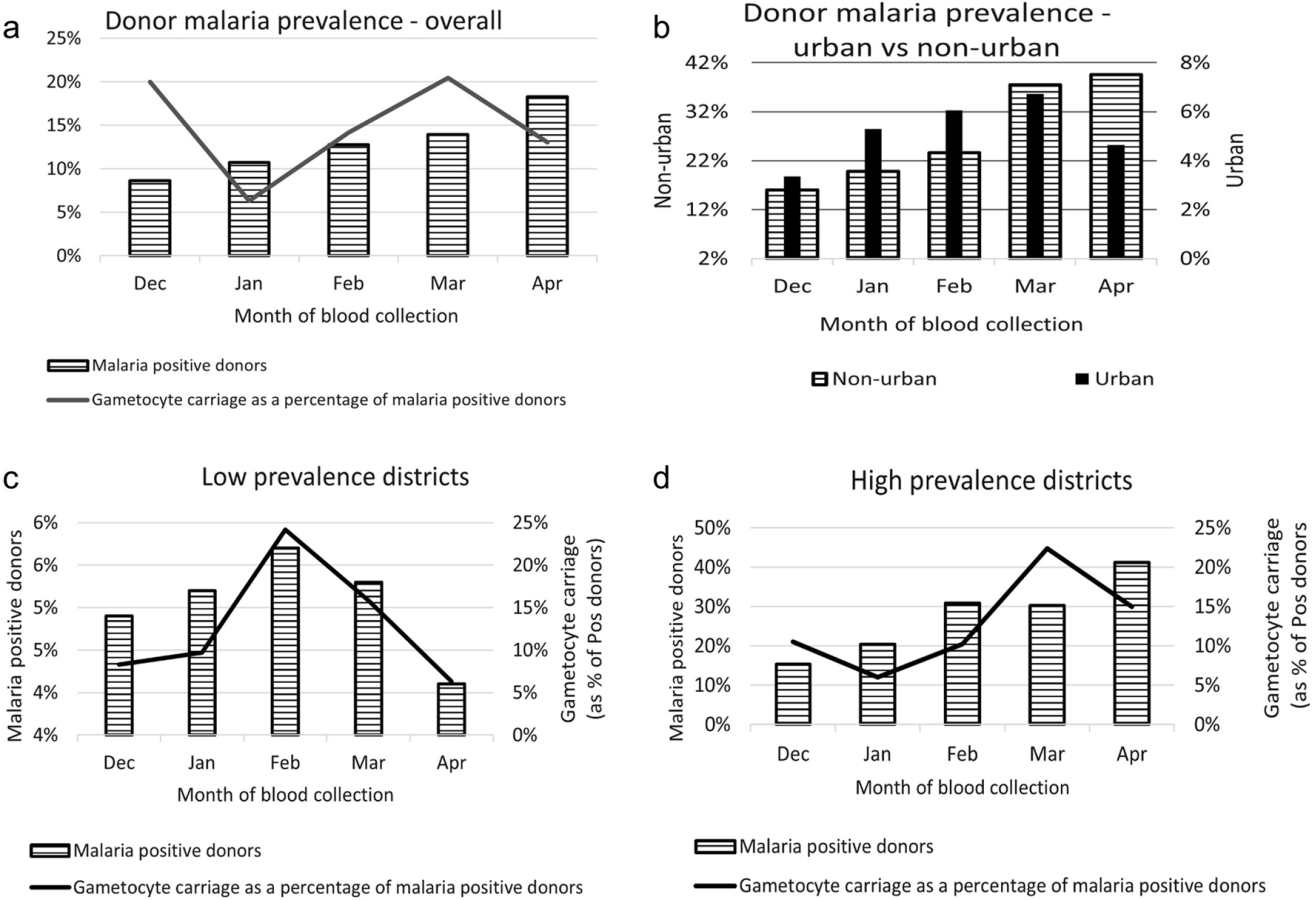
Donor malaria prevalence and gametocyte carriage (as a percentage of malaria positive donors) by month of blood collection (Dec 2019–April 2020) for **a** all districts combined **b** urban versus non-urban donors **c** low prevalence districts (< 10%) **d** high prevalence districts (≥ 20%)

**Fig. 3:**
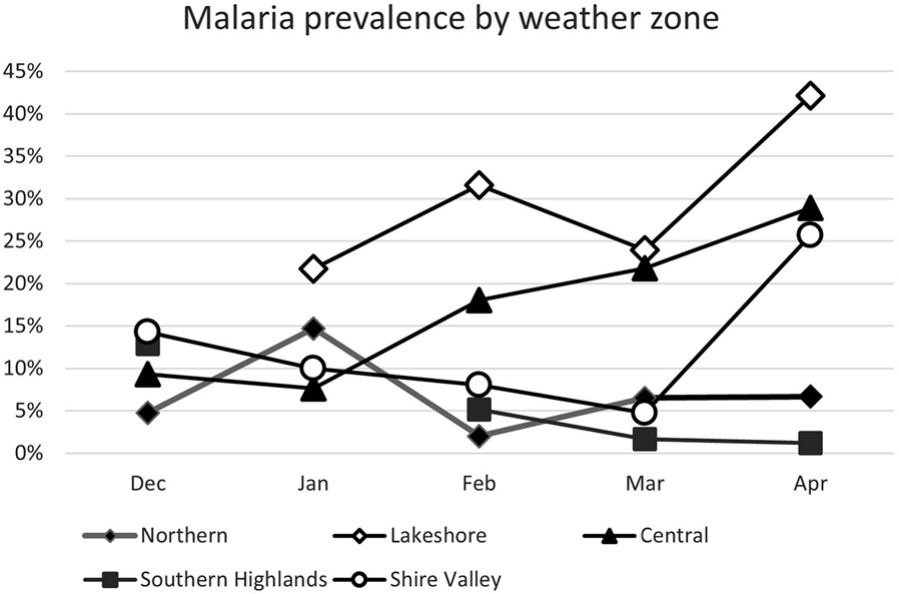
Donor malaria prevalence by month of blood collection grouped by weather zone. There were no donor samples from the Lakeshore region in December 2019 and from Southern Highlands region in December 2019 and January 2020

This secondary data analysis incorporated data from blood donor samples collected from December 2019 to April 2020. The trends of the monthly malaria prevalence averages for all districts; urban versus non-urban donation site locations; low prevalence zones (< 10% malaria positivity rate); and high prevalence zones (≥ 20% malaria positivity rate) are shown in Fig. 2 and by weather zones, in Fig. 3.

### Determinants of malaria prevalence

**Fig. 4:**
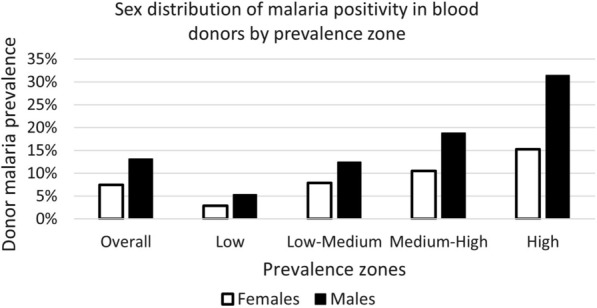
Malaria positivity in blood donors based on sex and prevalence zone. Overall, all districts; Low, districts with donor malaria prevalence  < 10%; Low-Medium; districts with donor malaria prevalence 10% to  < 15%; Medium–High; districts with donor malaria prevalence 15% to  < 20%; High; districts with donor malaria prevalence  ≥ 20%

The sex distribution of malaria positive donors overall and by prevalence zones is shown in Fig. 4. The overall malaria prevalence rate at 7.4% was significantly lower in female donors compared with 13.1% for males (p < 0.0001). These differences remained significantly different for both high (p < 0.0001) and low (p = 0.0327) prevalence zones but not for the intermediate groups, where donor numbers were fewer.

**Fig. 5:**
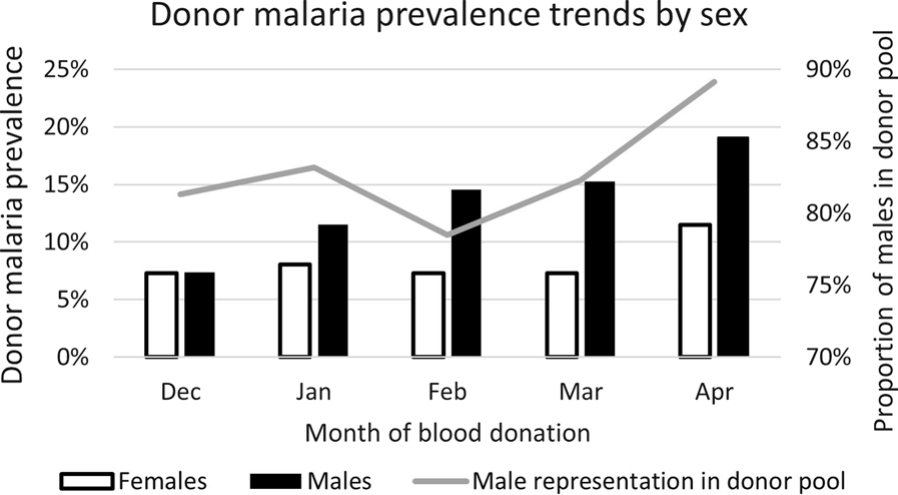
Donor malaria prevalence trends over time by sex. The proportion of male donors in the total donor pool per month of collection is shown by the grey line

To investigate what drives the difference between male and female donors, a multivariable regression analysis was undertaken to quantify differences of potential confounding factors such as age, urban vs non-urban location and month of collection in males and females (Table 2). For females, only location was statistically significant. Urban female donors were less likely to be malaria positive than non-urban female donors. In male donors however, location, age and month were all significant predictors of malaria positivity. Younger males were more likely to be malaria positive compared to older males, as were non-urban male donors compared to urban male donors. Interestingly, the individual month odds ratios for males showed a statistically significant positive trend (except for February), increasing from 1.51 in January to 2.48 in April. This seasonal trend was further corroborated by Fig. 5 which highlights that males mirrored the overall pattern of increasing prevalence from December 2019 through to April 2020, whereas females showed a more stable prevalence rate.

**Table 2:** Multivariable logistic regression analysis of malaria positivity in male and female donors

	Male donors			Female donors		
	OR	95% CI	p value	OR	95% CI	p value
Age	0.98	0.96–0.99	0.002	0.99	0.95–1.03	0.70
Location						
Urban	0.22	0.17–0.29	< 0.001	0.35	0.15–0.81	0.02
Month						
January	1.51	1.03–2.22	0.035	1.06	0.42–2.67	0.90
February	1.20	0.83–1.71	0.329	0.74	0.31–1.72	0.48
March	1.69	1.15–2.48	0.007	0.87	0.33–2.26	0.77
April	2.48	1.75–3.52	< 0.001	1.56	0.63–3.90	0.34

The reference group for location was non-urban and December for the month of collection

*OR* odds ratio, *CI* confidence interval

### Parasite burden and gametocyte carriage

**Fig. 6:**
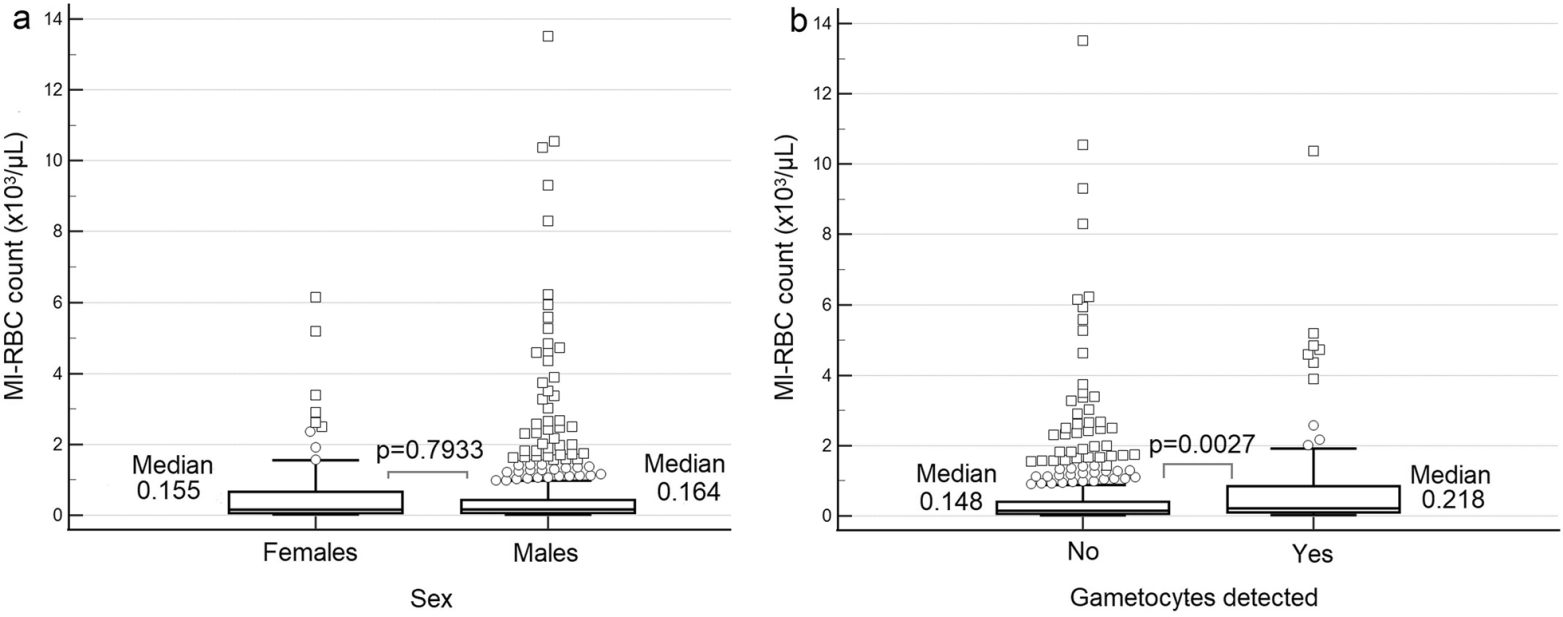
Parasite density distribution for malaria-infected donors based on gender and gametocyte carriage. **a** Parasite density based on gender, **b** parasite density based on gametocyte carriage. The boxplots highlight the respective age medians, lower and upper quartiles and mild (open circles) and extreme (open squares) outliers

There was no difference (p = 0.7933) in parasite density between male and female infected donors (Fig. 6a). The median MI-RBC counts were 0.164 × 10^3^/µL (IQR 0.066 to 0.440) and 0.155 × 10^3^/µL (IQR 0.057–0.626) for male and female donors respectively. There was also no difference (p = 0.9472) in gametocyte carriage amongst male (12.4%) and female (12.7%) parasitaemic donors. The median parasite density was however significantly higher (p = 0.0027) in donors in whom gametocytes were detected (MI-RBC 0.218 × 10^3^/µL, IQR 0.104–0.849) by the XN-31 analyser, compared with malaria-infected donors where gametocytes were not detected (MI-RBC 0.148 × 10^3^/µL, IQR 0.061–0.404) (Fig. 6b). There was also no difference in gametocyte carriage in low (12.9%) versus high (15.3%) malaria prevalence regions (p = 0.9472). Of the parasitaemic donors that harboured gametocytes, 5.3% (4/76) had gametocytes only (median MI-RBC 0.045 x 10^3^ p/μL), whereas 94.7% (72/76) had both ring forms and gametocytes (median MI-RBC 0.252 x 10^3^ p/μL). The median MI-RBC value differences for these two groups was statistically significant (p = 0.0023).

## Discussion

In 2020 the Sysmex XN-31 automated malaria detection analyser was evaluated at the MBTS, utilising samples from 5281 blood donors, collected nationally from 24 of the 28 administrative districts in Malawi [9]. In this current study, an in-depth analysis of the same dataset was conducted utilizing donor demographic and blood donation site location to explore the feasibility of using blood donor malaria screening as a complementary source of malaria surveillance data.

For malaria elimination to succeed, it is essential that countries generate and use high quality surveillance data to accurately define malaria incidence over time and space [15]. Depending on availability of quality data, the WHO uses two different methods to estimate malaria burden for its annual World Malaria report [1]. The first method estimates malaria incidence from reports of malaria cases compiled by national health ministries. These data are then statistically adjusted for reporting completeness, positivity rate among suspected cases, and the rate at which febrile patients seek public healthcare. However, in many malaria-endemic countries (about 30 Sub-Saharan African countries), precise estimation of malaria burden is hindered by data scarcity due to weak surveillance systems [16]. As a result, the WHO has resorted to geospatial epidemiological models to estimate malaria burden in these countries [1]. These models rely on asymptomatic parasite infection prevalence data collected largely from children under the age of 5 years, via infrequent, costly, and logistically complex surveys.

The most recent Malawi MIS took place in 2021, and prior to that in 2017 [17], and 2014 [18]. The MIS tests children aged 6–59 months for anaemia and malaria parasitaemia. Malaria prevalence in this age group was 10.5% in 2021 (unpublished 2021 Malawi Malaria Indicator Survey), a significant decline from 24% in 2017. Many individuals, including children, as demonstrated in these household surveys, are parasitaemic without showing any signs of infection. Besides placing infected individuals at risk of developing anaemia and associated morbidity, asymptomatic infections play an integral role in sustaining transmission in regions where malaria is endemic as they largely outnumber symptomatic infections [19]. Systematic reviews of studies conducted in malaria-endemic countries have shown that asymptomatic malaria is common in healthy adult blood donors, and that the prevalence of malaria-infected donors generally mirrors the overall malaria burden of the region [8, 20].

The overall donor malaria prevalence rate in this study was 11.6%, increasing progressively from December (8.6%) to April (18.3%) (Fig. 2a). This seasonal trend is in line with what has been previously reported to be driven largely by the annual rains that typically begin in November–December and last through March–April in most parts of the country [21]. However, when viewed by weather zones (Fig. 3), this trend was only observed for the high prevalence Central (17.1%) and Lakeshore (27.8%) zones, with the latter having the highest prevalence rate in Malawi, which has been consistently reported [22, 23]. In contrast, the low prevalence Southern Highlands (6.1%) zone, showed the opposite, with a consistent downward trend with the lowest donor malaria prevalence in April, whereas the Northern (7%) and Shire Valley (12%) zones had variable patterns. These data are consistent with those of others [17, 18, 24] with the Central administrative region having the highest and the Northern region the lowest malaria prevalence rates (Fig. 1b), but the same does not hold true for the Southern region. This region had a malaria donor prevalence below that of the Northern region in contrast to that of the 2015–2016 Malawi Demographic and Health Survey (which assessed parasitaemia using PCR in adolescent and adult males and females) [24] and the 2017 MIS [17], which reported high malaria prevalence rates for the Southern region, close or equal to that of the Central region. Whilst the absolute prevalence rates of the current study and these two Malawian surveys [17, 24] are not comparable, because of temporal (year and month/s of collection) and test group differences (children versus adults) and differing sensitivities of diagnostic test used for parasitaemia assessment (PCR > XN-31 > microscopy/rapid diagnostic test), the patterns observed are informative, and reflect similar trends. As these study data were obtained 3 years after the 2017 Malawi MIS, it is proposed that these changes reflect rapidly changing malaria dynamics in Malawi [25], rather than unreliable data. When comparing the 2014 [18] and 2017 Malawi MIS data [17], the overall prevalence of malaria in the under 5-year age group dropped from 33 to 24%, but the rate of change was highly variable amongst the regions. The Northern region had a 68% decline (29% to 11%), in contrast to more modest reductions of 28% and 21% respectively for the Central (36% to 26%) and Southern (33% to 26%) regions. It is thus conceivable that the low malaria prevalence finding in this study for the Southern region (7.9%), more in keeping with the Northern (8.6%) rather than Central region (18.5%), reflect a regional acceleration of decline here, in line with what was observed for the Northern region between 2014 and 2017 [17, 18]. This view is supported by the preliminary data from the still unpublished 2021 Malawi MIS report which indicate a significant decline in malaria parasitaemia in the 6–59 month age group, with the Southern region showing the greatest reduction from 26% to 10% (62% decline), and overall from 24% to 10.5%, in the 4 year period since the last survey. The 2021 MIS overall prevalence rate of 10.5% is not dissimilar to the 11.6% blood donor malaria parasitaemia prevalence in the current study (where 85.6% of samples were collected in 2020, and the remainder in December 2019).

These observations highlight that the episodic nature and short duration (1–2 months) of data collection utilizing epidemiological household surveys, and the delay in such results becoming available, makes accurate tracking of rapid, year-to-year changes in malaria burden a difficult task [16].

The current study data concur with the generally reported higher malaria risk associated with non-urban areas [26, 27]. They are also in keeping with previous Malawi survey findings [17, 24], notwithstanding that weighting was not applied and the definition of urban versus non-urban was crudely based on blood donation site location and not place of residence, and cognisant that non-urban versus urban malaria risk association in Malawi is more complex than simply considering physical location [28].

The study blood donor pool was 80.2% male with 95% of donors between 16 and 47 years. Female donors were slightly younger (median age 19 years) compared with males (median age 21 years). Urban donors comprised 45.9% of the pool with an even stronger male representation (90.2%), and significantly (p < 0.001) older (median age 25 years) compared with non-urban donors (median age 20 years). This urban male dominance and older age, accounted for the age difference between male and female donors overall. Parasitaemia rates were significantly (p < 0.001) and consistently higher in male donors (13.1%) compared with female donors (7.4%), irrespective of location or month of collection. This male bias of parasitologically confirmed malaria infection prevalence is in keeping with several other studies conducted in Africa [24, 27, 29, 30]. Interestingly, different temporal trends for male and female donor malaria prevalence were observed, with males mirroring the generally reported seasonal increase from November to April and female donors having a more stable malaria prevalence over time (Fig. 5). A study involving intensive follow-up of *P. falciparum* infections in Eastern Uganda [31], utilizing ultrasensitive molecular testing to distinguish new from persistent infections, demonstrated that whilst the incidence of new infections was the same in both sexes, males had a slower clearance of asymptomatic infections attributed to biological sex-based differences as no behavioural differences were recorded. Behavioural differences that lead to increased exposure to mosquito bites in men can however not be excluded in the current study. In contrast to asymptomatic parasitaemia, the incidence of clinical malaria appears to be higher in females [29] with pregnancy-associated loss of malarial immunity [32] and increased health-seeking behaviour in females [29] playing a contributory role. It is thus speculated that the observed increasing prevalence of parasitaemia in male donors only could be explained by the slower clearance of parasites in males compounded by the growing incidence of new infections as the seasonal changes become more conducive to promoting mosquito breeding sites and thus increased malaria transmission. Also, the reported increased incidence in symptomatic infections in females [29], would result in self-deferral from blood donation or rejection through health questionnaire screening at the donation sites. Furthermore, the enhanced health-seeking behaviour of females [29] would increase treatment-induced clearance of parasites in those diagnosed with malaria. Taken together with the biologically faster parasite clearance in females [31], these factors may be a plausible explanation for the relatively stable malaria parasitaemia prevalence observed over time in the female donors in this study. The peak in April, observed also in the female donors, may well be the compounded effect of a marked increase in incidence driven by rainfall and humidity. Whilst parasite rates differed amongst the sexes, the parasite burden (MI-RBC count) in infected donors did not reach significance (p = 0.7933) in keeping with what has been reported elsewhere [31].

For surveillance data to be translated into meaningful interventions and to monitor their impact, it needs to be representative of the community at risk of malaria. Sample size determination and sampling for a typical MIS is a highly complex process, and whilst the bigger the better, it is a balancing act between the demands of the analysis, implementation team capacity and budget constraints [33]. The 2017 Malawi MIS [17] sampled 3750 households, representing 0.13% of total households, and tested 2031 children under the age of 5, representing 0.08% of children in this age group, based on the 2018 Malawi population and housing census data [14]. In the current study 5281 blood donors (aged 15–64) were tested, representing 0.06% of this age group (using the same census data) for both sexes combined, and 0.1% and 0.02% males and females respectively. There was also a reasonable match between donor distribution by region and population density. The split of donors by the Northern, Central and Southern regions gave a proportion of 9%, 39% and 52% respectively, compared with 13%, 43% and 44% for the total population of the respective regions. The disproportionate representation of the regions is best explained by the non-random nature of sampling due to preferential inclusion of blood donor samples from low workload days, as the original study was not designed with a surveillance data collection objective in mind. This may also account for the proportional over-representation of urban donors relative to the urban population size (urban 0.13%, non-urban 0.04%). Only ~ 25% of the total number of donations received during the study period were included, as study-related sample processing was restricted to low workload days. If XN-31 blood donor screening had been integrated into MBTS in 2018 a total of 66931 [34-37] individual donor blood samples would have been screened for malaria, which represents 0.73% of the total population aged 15 to 64 years. Stratified by sex and urban/non-urban location utilizing the current study donor pool distribution, all sub-groups would have greater representativity (ranging from 2.97% for urban males to 0.23% for non-urban females) than the MIS. In this regard routine malaria screening of blood donors would serve as an excellent data source to complement traditional household surveys. Notably, the XN-31 is a high throughput automated analyser which could be easily integrated into blood transfusion services for the testing of subjectively healthy asymptomatic adolescents and adults to provide real-time information on the trends of the size, location, age, and gender distribution of the asymptomatic malaria parasite reservoir to complement existing malaria surveillance activities. Furthermore, M’baya et al. [9] have demonstrated that XN-31 has superior sensitivity compared to routine microscopy for malaria screening in blood donors. As screening and data collection would be a continuous activity, the analysis of trends which would have a comparable baseline, more so than absolute values, would be of most value to inform malaria control interventions. Furthermore, as blood transfusion is a core life-saving intervention globally, blood donation is a vital activity undertaken in all communities. The source of “survey” participants is thus self-selected and in continuous supply. Notably also, blood donor surveillance data would be highly representative of males, a group currently excluded from traditional household MIS, yet with their greater persistence of parasitaemia and thus higher parasite rates, are an important source of ongoing malaria transmission. Recognizing both the value and limitations of periodic household surveys, the need for complementary data, for example school-based surveys [38] or from antenatal clinics [39], has been highlighted by others. School-based surveys, whilst targeting a unique population would still be limited by their episodic nature, whereas antenatal clinic screening may provide continuous data.

Besides providing quantitative parasite counts, the XN-31 analyser also provides information on the presence of gametocytes, as a research-use-only parameter. Gametocytes were observed equally in malaria-infected male and female donors, with the parasite burden (MI-RBC count) being significantly higher in gametocyte carriers compared with malaria-infected donors where gametocytes were not detected (Fig. 6) in line with what has been previously reported [40, 41]. Interestingly, donors in whom only gametocytes were detected had significantly lower MI-RBC counts than those with ring forms and gametocytes. The gametocyte carriage, as a percentage of malaria-infected asymptomatic donors, was not statistically different between high and low prevalence regions. Monitoring trends of gametocyte carriage is an important component of surveillance as it provides insights into the pool of individuals capable of onward transmission [42].

This study has limitations. Firstly, the original study was designed exclusively to confirm analytical performance of the XN-31 analyser as a malaria diagnostic test, hence the focus was on inclusion of a sufficient number of blood donors over a certain time-period with no regard for proportional representativity of donors based on districts (blood collection site), urban versus non-urban location nor month of collection. Consequently, reliable parasitaemia prevalence rates could not be established for administrative districts with too few or no donor inclusions in this study nor could weighting be applied to adjust for this disproportional representation. Also, only donor samples received on low workload days (~ 25% of total donations during the study period), were included, meaning that the study sample composition may differ from the complete donor pool. Furthermore, the data collection did not span a full year hence it does not cover the full seasonal range of malaria transmission. Topazian et al. [24], using quantitative PCR testing, showed that 55.6% of malaria-infected individuals identified in the 2015–2016 Demographic Health Survey had parasitaemia of  ≤ 10 p/µL. It is thus acknowledged that a substantial number of malaria-infected blood donors would have very low level parasitaemia falling below the limit of detection of the XN-31 (20 p/µL). PCR is however not an accessible diagnostic tool, because of cost and complexity, and thus rapid diagnostic tests and microscopy are widely utilized for malaria surveillance parasite screening. As shown in the original blood donor screening study at MBTS [9], XN-31 detected almost twice as many malaria-infected donors compared with microscopy. In this regard, although lacking the sensitivity of PCR, the XN-31, due to its automated nature, superior sensitivity to routine microscopy, and ease of use, is an excellent diagnostic tool for malaria screening within blood transfusion services, and thus ideal for generating complementary malaria surveillance data, where the focus is on the observation of trends. In addition, the populations surveyed in this study (blood donors) and in the MIS (children under the age of 5 years) are different, and thus not directly comparable. However, malaria screening of blood donors brings a different dimension to malaria surveillance since it targets the detection of the asymptomatic reservoir of parasites in adults, an important metric for malaria control strategies. Furthermore, blood donation is an ongoing activity, and thus implementation of XN-31 malaria screening of donors, would generate data continuously in real-time, allowing the rapid detection of changing trends which is not possible with episodic household surveys. Also, as blood donors are recruited across a wide geographic location, this facilitates a high spatial resolution which is currently only obtainable from clinical case reporting. Thus, XN-31 blood donor screening would provide an easily accessible source of complementary malaria surveillance data, since this secondary data analysis has shown that the malaria prevalence in blood donors mirrors the patterns and trends detected by other means of surveillance in Malawi.

## Conclusions

Blood donors in malaria-endemic countries are an untapped source for the systematic measurement of the asymptomatic parasite reservoir. The XN-31 detects malaria with high diagnostic accuracy and provides parasite burden quantification. Thus, the XN-31 automated analyser is an ideal tool for malaria screening of blood donors, which could not only contribute towards increased blood safety, but also serve as a complementary source of surveillance data for malaria control. Blood collection is a vital healthcare activity in all countries, and perhaps more so in malaria-endemic countries, such as Malawi, where malarial anaemia [43] is a key driver of blood transfusion demand. Furthermore, blood donations are sourced countrywide, all year round, even during pandemics. The blood donor pool can thus be considered as representative of the population. Lastly XN-31 also provides an indication of gametocyte presence and a haemoglobin value with each measurement, both of which are important metrics for malaria surveillance.

## Data Availability

The datasets used and analysed during this study are available from the corresponding author on reasonable request.

## Abbreviations

CI: Confidence interval
GTS: WHO global technical strategy for malaria
IQR: Interquartile range
MBTS: Malawi Blood Transfusion Service
MI-RBC: Malaria-infected red blood cells
MIS: Malaria indicator survey
OR: Odds ratio
PCR: Polymerase chain reaction
p/µL: Parasites/microlitre
RBC: Red blood cell
TTM: Transfusion transmitted malaria
WHO: World Health Organization
